# Human rights’ interdependence and indivisibility: a glance over the human rights to water and sanitation

**DOI:** 10.1186/s12914-019-0197-3

**Published:** 2019-03-08

**Authors:** Priscila Neves-Silva, Giselle Isabele Martins, Léo Heller

**Affiliations:** René Rachou Institute - Fiocruz Minas, Augusto de Lima Avenue, 1715 CEP: 30, Belo Horizonte, Minas Gerais 190-002 Brazil

**Keywords:** Human rights, Water, Sanitation, Homeless

## Abstract

**Background:**

In 1948 the Universal Declaration of Human Rights was drawn up. The content of this document was further reflected two treaties, the Covenant on Civil and Political Rights, and the Covenant on Economic, Social and Cultural Rights. To try to maintain the interrelationship between the rights contained in each document, the idea that all rights are interdependent and indivisible was stressed. Based on this vision, this study aimed to explore the extent to which the violation of the human rights to water and sanitation interferes with the guarantee of other rights, addressing the principles of interdependence and indivisibility.

**Methods:**

For that, 24 homeless, in the city of Belo Horizonte, Brazil, were interviewed. Individual and group interviews were carried out in addition to participant observation. The content analysis was used in order to analyze the data collected.

**Results:**

The research found that violation of the rights to water and sanitation promotes violation of other rights, such as health and education rights, strengthening the view of rights’ interdependence and indivisibility.

**Conclusion:**

It is important to affirm that the protection of human rights must be consolidated at an operational and normative level, aligned to concepts of indivisibility and interdependence as it has been proposed for approximately seven decades.

**Electronic supplementary material:**

The online version of this article (10.1186/s12914-019-0197-3) contains supplementary material, which is available to authorized users.

## Background

Last year, seven decades of the adoption of the Universal Declaration of Human Rights, the main document that underlies the elaboration of several international treaties and covenants as well as serving as a model for many national constitutions, were completed. Adopted in 1948 by 48 countries and reiterated in 1993 through the Vienna Declaration of Human Rights signed by 171 countries, it remains the basis for many international agreements.

The drafting of the Universal Declaration of Human Rights gave rise to two main Covenants, the International Covenant on Civil and Political Rights; and the International Covenant on Economic, Social and Cultural Rights. The first one consists of a set of rights that aim at individual freedoms and procedural guarantees of access to justice and political participation. On the other hand, the second group brings together the rights related to the state of social welfare, for which the State must intervene to ensure a more fair and egalitarian society [1, 2].

Despite of having two covenants, human rights must be seeing as a system where all rights are interdependent, indivisible and interrelated. According to Minkler and Sweeney [3] the vision of a comprehensive human rights system was built on the understanding that, in order to guarantee the dignity of the human person, all human rights should be reached. In this way, the integral human rights system is established where the violation of one of them, either civil or political right; or economic, social and cultural, damages the achievement of the others.

In the following decades, the main United Nations General Assembly resolutions reaffirmed the vision of the interdependence of human rights – “All human rights are universal, interdependent and interrelated. The international community must treat human rights globally in a fair and equitable manner, on equal terms, and with the same emphasis.” [4] - and the Vienna Declaration, renewed and expanded this understanding by stating that there is interdependence between human rights, democracy and development.

However, the indivisibility of human rights is a contested point on which scholars disagree. Neier [5], Roth [6], Nickel [7] challenge the effectiveness of economic, social and cultural rights, and point out that a very large number of human rights make it difficult to implement, making it almost impractical. However, authors such as Feinberg [8], Copp [9] and Sen [10, 11] point out that the understanding of human rights as essential to provide the basic needs of individuals and guarantee freedom, justifies the role of economic, social and cultural rights. In this scenario, Minkler and Sweeney [3] point out that until this issue is solved, researchers should focus on the human rights considered to be basic.

According to Payne [12] the basic rights, defined by Shue [13], would be those that ensure self-respect and survival, and are therefore necessary for the guarantee of all others. For this author there were two types of basic rights: the rights related to security and the ones related to subsistence. Security rights refer to civil rights as those that protect the human being from torture, rape, assault and death. Subsistence would correspond to economic, social and cultural rights such as access to water, food, clothing, shelter and health care. Thus, when taken together, these rights would be mutually indispensable and equally necessary for the guarantee of other rights. Therefore, all other non-basic rights would rely on these rights.

Some authors argue that the interdependence and indivisibility of human rights will be ensured when the most vulnerable, socially excluded and marginalized populations are included [2, 14]. For them, the economic, social and cultural rights are more addressed to this population, favoring the social inclusion and the construction of a more egalitarian society. Thus, to neglect these rights would be a reflection of societies marked by injustices and social disparities.

Within this scenario, one finds the Human Rights to Water and Sanitation (HRWS), recognized by the United Nations Assembly in 2010, through resolution A/RES/64/262 [15], which are closely related to the right to a dignified life. The right to water is described in General Comment N° 15 [16], adopted by the Committee on Economic, Social and Cultural Rights, and affirms that it is the right of all, without discrimination, to access safe water for drinking, cooking and hygiene of the home and belongings. In 2015, after understanding that sanitation has distinct characteristics that specify it, and that when separating it from the water it would be possible to give greater visibility to its peculiarities, it became an independent right. Thus, resolution A/RES/70/169 defined sanitation as a separate but integrated right to the human right to water.

General comment number 15 points out the normative content of access to water and defines that the access to this resource should be within the human rights framework, therefore, it is necessary to ensure availability, quality and safety, accessibility, affordability and acceptability. For sanitation, hygiene, privacy and dignity must be ensured as well, as attested by the Special Rapporteur on the Human Rights to Water and Sanitation [17]

The JMP report, published in 2017, which has been monitoring global progress on access to water and sanitation, showed that 844 million people lack access to basic water and 2.3 billion lack access to basic sanitation. Vulnerable populations are the ones that suffer more from the lack of those services [18]. It is necessary to remember that 193 Member States from United Nations, including Brazil, signed the Sustainable Development Goals. The goal 6 meant to achieve universal access to water and sanitation for all. By 2030 no one should be left behind [19].

The homeless live in a vulnerable situation and do not have access to a minimally adequate housing [20]. The United Nations estimates that, in the world, 100 million people are homeless [20]. In Brazil, it is believed that this population is around 101 thousand [21].

Walter’s [22] study on access to water and sanitation for homeless in India verified that these people have the HRWS violated, affecting, among other things, their health and dignity. For this author the reach of the HRWS for this population goes beyond technical questions demanding an understanding of how the vulnerability situation of these individuals was created and is maintained, in this way, these people would be exposed to structural and social iniquities that include the violation of rights.

These findings are aligned to Udin et al. [23] who carried out a survey in Dhaka, Bangladesh, and concluded that the lack of access to water is related to a marginal position in society, preventing the access to basic services such as housing, education and health, besides water and sanitation. For these authors, this lack of access reflects an asymmetric distribution of power and opportunity, and vulnerability is a result of the inequities in power relations that would lead to rights’ violation. Hence, these individuals would face difficulties accessing subsistence rights, but also claiming them. In this scenario, the authors draw attention to the importance of the concept of indivisibility of human rights. For them, the guarantee of HRWS should be rooted on the concept of a right-based approach, which broadly acts in all the rights that are mostly denied to vulnerable and marginalized people.

Neves-Silva and Heller [24], evaluated homeless’ access to water and sanitation in Belo Horizonte, Brazil, and verified that the lack of these accesses implies, among other things, in health problems, physical and psychological violence by the society and public administration, mainly municipal officers, and the risk of violence against women. In addition, it has been found that the normative content of both the right to water and sanitation is generally not respected, as are human right principles such as non-discrimination, access to information and participation. These findings reinforce the importance of discussing HRWS using the concepts of interdependence and indivisibility.

Therefore, based on the idea of a comprehensive human rights system and the importance of access to economic, social and cultural rights by the marginalized population with a view to establishing a more egalitarian society, this article aims to explore the extent to which violation of HRWS of homeless population at Belo Horizonte, Brazil, interferes with the guarantee of other rights, addressing the principles of interdependence and indivisibility among them.

## Methods

This is a qualitative research with homeless living in the city of Belo Horizonte. The qualitative methodology was chosen in order to comprehend better the reality in which the homeless live, and to get their perspective about it [25].

### Description of study area

Belo Horizonte is the 6th most populous city in Brazil. The 2017 Population and Household Census indicated that its population is 2. 523. 794 [26]. It is located at the southeast and it is the fourth wealthiest city in the country. The main economic activities are services like financial services and trade. Nevertheless, the homeless population in the city is growing and data from the Third Census on the Homeless Population and Migrants, carried out in 2013, showed that there were 1827 homeless people in the city; an increase of 57% compared with the 2005 census [27]. It is estimated that at the end of 2017, 4.500 people were living in the streets [28].

### Selection of participants

Participants were selected according to a criterion of accessibility, after participant observation. Approaches in the street took place in the central region of the municipality, which concentrates the largest number of homeless [27]. The approaches were made introducing the researchers and the purposes of the research. Some approached people were suspicious and did not want to participate or were drunk and/or drugged, and it was not possible to interview them. The saturation sampling technique, in which the absence of new themes and the repetition of content in the interviews are indicative that the main ideas have already been raised, was used to define the number of interviewees [29].

In order to carry out the group interview, a contact was initially made with the Pastoral de Rua de Belo Horizonte, an organization linked to the Catholic Church, acting to promote dignity to homeless, and that has been operating in Belo Horizonte since 1987., who promptly received us and pointed out that they were not aware of any other research that had approached homeless and the access to water and sanitation. Every week, at the Pastoral’s premises a meeting takes place along with the homeless. Researchers attended those meetings for 2 months and presented the research to the group, which was very interested.

### Data collection

Individual interviews and one group interview were used to gather data, those two techniques were used as complementary. This helps to better understand the reality [25]. These interviews, carried out from May through July 2016, followed a semi-structured script, elaborated from the researchers’ experience and literature review, and contained general questions about street life, questions about the normative content of HRWS (availability, quality and safety, accessibility, affordability and acceptability), and issues that addressed some of the principles of human rights (non-discrimination, information/transparency and social participation) (Additional file 1).

Initially, a pilot interview was carried out to assess the script and any question that were not well understood by the participant were modify. After the necessary changes were made, researchers went to the field. On the first day in the field, a participant observation [19] was made in a public space downtown where many homeless live. The choice of this square is due to the high number of homeless that pass through or live there and the presence of a water source. During the participant observation, made in two days, the two researchers assessed how homeless used the water source located in the square and how was the routine of the people who lived there. The participant observation was essential to have a better understanding about the ways this group uses water and sanitation services.

Men and women living in the streets, aged > 18 years old, participated in the study. Twelve people were interviewed individually, out of which, five were women, and the interviews were carried out on the streets or in the premises of the Pastoral de Rua. The group interview was carried out at the Pastoral’s premises and included twelve participants, out of which, four were women. The 24 participants in the study were aged between 20 and 55 years old and the time elapsed since they became homeless ranged from 15 days to 30 years.

### Data processing and analysis

The interviews were recorded, transcribed and analyzed by the two researchers that collected them. The technique of content analysis was used [30], through which the information collected was systematized into thematic categories. This involved reading through participants responses and assigning codes to specific aspects of the responses.

In order to reduce the influence of the researchers’ own backgrounds, conceptions and perceptions of the topic during the interview and the analytical process, the interviews and participant observations were done by the two researchers. Both of them had a field book, and all data analyses were done by the two researchers independently and then compared.

A code book was then developed, discussed and accepted by the researchers. Then, the codes were grouped into themes: the discrimination due to lack of access to water and sanitation; the lack of access to education and health; the lack of access to leisure activities; the lack of social participation; the lack of access to information. Respondent statements, which are especially expressive, informative and representative, were selected and are presented as quotes.

### Ethical considerations

Gathered data is confidential and the anonymity of the participants has been guaranteed, so that their names will not be unveiled in any situation. Participants that were individually interviewed were identified with the abbreviation HL (Homeless) followed by the serial number in which they were interviewed. Those who participated in the group interview were identified as EG/HL followed by the participation order number. Participants were informed about the project and invited to participate voluntarily, signing the Informed Consent Form. They were certified that they could feel free to leave the study at any time. The survey herein was approved by the Research Ethics Committee from the René-Rachou Research Center – under protocol CAAE 49209515.0.0000.50.91.

## Results

### The discrimination due to lack of access to water and sanitation

Lack of access to water and sanitation results in discrimination and more difficulty to access water. The dirty person is not allowed to enter in an establishment to ask for water and also are treated as garbage by society. Therefore, the participants pointed out that lack of access to water and sanitation services, and the association society makes between them and rubbish, promote the construction of the stigma and strengthen their marginalization and exclusion. The exclusion and marginalization suffered by them result in lack of access to many places, and in many circumstances they are like invisible to other people.

“Stinky homeless entering on an establishment asking for a glass of water? Come on! There’s no purpose on that.” GF/HL 3.

“When you are homeless and arrive all dirty, you are hardly looked at. People do not pay attention at you.” HL 7.

“The society sees the homeless as a burden, someone who litters the city.” HL 8.

“We have to remove the garbage! Get out garbage.” HL 5.

### The lack of access to education and health

Through the reports of the homeless, it is possible to see that the absence of HRWS has repercussions on rights’ guarantee, such as education and health. As they are seen as dirty, stinking and using dirty cloths, they feel bad going to school and staying in the classroom. Therefore, as access to education is, in many cases, limited, they will face many barriers to create opportunities to improve their lives.

“This is the second time I try to go back to school, but I was ashamed because I couldn’t get the money to take a shower so not to arrive stinking at school.” HL12.

They also face some difficulties when looking for health services. Health professionals, in some cases, ask them to take a shower or change clothes before the appointment. As they have difficulties in finding places that allow them to take a bath or clean their belongs, they avoid going to the health care center.

“When he/she gets sick and looks for a hospital, it’s not everyone who likes to take care of him/her, either … he/she has to be clean to be taken care of, if he/she gets dirty, is looked at with an ugly face.” GF/HL7.

“If homeless gets sick, he/she goes to the hospital but he/she is not admitted because he/she got there dirty; you have to hunt down a place to take a shower, and where are you going to take a shower? At the bus station if you have no money you do not take it. In the spout, for you to leave here and go there to take a shower and then come back to the hospital, you have already missed the appointment. At the Station Square, if you take a bath in the fountain, the water is returnable and you will get skin diseases. If you go to take a shower in another corner, by the time you manage to come back to the hospital, the doctor will say, “your record has already been canceled” and you stay ill. That’s the way it is.” GF/HL3.

### The lack of civil and political rights

As homeless are seen as dirty and their cloths and belongings with a bad smell, they are discriminated and this affects the way they are treated by society. Some state officials, in special the police, are not prepared to deal with them and their particularities, necessities and way of living. This results in approaches done with violence and inhumanity. Theirs belongings are taking off and the police usually take their documents which prevent them to vote. This affect the access to the right to vote and the enjoyment of other political and civil rights, as they have problems to get their documents.

“They try, in all ways, to oust homeless, taking our belongings, our documents.” HL 5.

“I’ve been trying to get my documents for four months. The police carry my belongings; no one wants to help homeless, they rather undermine us.” GF/HL 2.

“At the elections I do not vote.” HL 4.

### The lack of access to leisure activities

The lack of access to water and sanitation also prevents homeless from participating in social life and leisure activities. They are not allowed to get into public places as municipal theater or central market, and, sometimes, they are removed from public spaces when there is a special event in that area. For them, they are excluded because they are dirty and not smelly.

“A homeless cannot get into the Palácio das Artes (theater in the city). If he/she is dirty, he/she is not allowed to enter the Palácio das Artes, if he/she is dirty, he/she cannot enter the Central Market.” HL 5.

“If a party is thrown at the Station Square, homeless are removed from the area. They are not allowed to stay near the party. To enter you must be well dressed, be clean and smelly.” HL 8.

### The lack of access to information

Also, they cannot participate actively in decision make process as they do not have access to information and they feel that they do not have rights. As they are seen as dirty, and wearing dirty cloths, they are compared to trash, they are invisible and they are not part of the society. For these reasons, they are not informed, and, in many cases, not allowed to participate in meetings to discuss public policies. This reinforces discrimination and put them away from decision make processes, which helps to increase the feeling of having no rights.

“The government never calls, and will never call, homeless to discuss the water issue. We are garbage, the cancer of the world. When publicized, it is only done among higher classes, we the street class, we are invisible and have no access. We are the untouchables. Society sees us and runs away: "no, it is full of animals, garbage out there”. HL 5.

“I’m nothing; I have right to nothing.” GF/HL 3.

## Discussion

These reports are aligned to what many authors point out: the full rights’ accomplishment must be approached from the indivisibility and interdependence point of view. Failure to implement some human rights can cause harm to others, in which case it is important to check the damages and risks that can be created when certain rights are not guaranteed. The relationship between access to water and sanitation and the accomplishment of other rights is evident when homeless report that the lack of these services often prevents them from attending public establishments.

Nickel^(7^^)^ argues that indivisibility and interdependence are not the same thing since indivisibility supposes a much stronger form of interdependence, in what it claims a bidirectional support, where the guarantee of a right would only exist if the other is implemented, and vice versa, ensuring a reciprocity in the relationship. The author understands that interdependence occurs when one right depends on another to be guaranteed but there would be no reciprocity, that is, one right would be dependent on another right, but this other right would not need the first to be guaranteed, with that there was no bidirectional support, it would be unidirectional. Thus, for this author, several rights are interdependent, but they are not indivisible.

Nevertheless, one can say that HRWS, in the context herein evaluated, fall within the two concepts, interdependence and indivisibility. This is because the lack of access to water and sanitation echoes in the violation of other rights, such as health and education. On the other hand, the lack of access to education, health and housing can result in HRWS violation. Failure to accomplish education and health rights, especially education, prevents homeless from re-entering society, what would improve access to water and sanitation, housing and other economic, social and cultural rights, as well as of politic and civil ones. Therefore, one can say that there is a strong and bidirectional relationship between these rights, so they are interdependent and indivisible. For this reason, one can say that the quality of implementation of HRWS interferes in the quality of implementation of the human rights framework.

In addition, when it comes to HRWS, it can be said that it is a subsistence right, since its recognition is anchored in the right to a quality life, and Shue [13] understands livelihood rights are indispensable for the accomplishment of many other rights.

The study also shows how violation of HRWS promotes violation of civil and political rights, showing that HRWS interdependence with other rights is a reality that should not be neglected. Besides, violation of the HRWS also inhibits the active participation of homeless people in society. They can’t participate in leisure activities and have difficulty accessing public places.

This finding coincides with Kaufman’s [31] study, in which he assesses the interdependence and indivisibility between the so-called first generation (civil and political) and the second generation (economic and social) rights, and finds that they are interrelated. However, it challenges the findings of Leblang et al. [32] that found a negative correlation between livelihood and security right.

The HRWS violation, also, helps to strengthen the stigma and prejudice that put these people at the margins of society. As reported, they are seen as junk, as dirt, as something that can be disposed, that promotes the loss of self-esteem and favors social exclusion.

Quane [14] states that besides being considered indivisible and interdependent in what refers to implementation, it is important to understand that rights are also interdependent and indivisible in their content. For this, the author draws attention to the right to participation. Everyone can invoke direct participation in what refers to “the right to effectively participate in public life” [14], but the right to participation is embedded in several other rights, such as in HRWS, and is more generally represented in the covenants. Thus, for the author, the boundaries that existed between right contents were undone and instead, the degree of interdependence and indivisibility was broadened and no longer confined to a specific covenant, but reflected a trend of the international protection system of the process of interaction between human rights.

However, how to you claim for these rights when individuals are barred from entering public spaces due to lack of bathing or clean clothes and belongings? How to assert the right to participation if they cannot access information?

The full exercise of political rights presupposes an active participation of the subjects and promotes the empowerment of the most vulnerable populations, improving their capacity for pressure, articulation and mobilization, aiming at a better implementation of economic, social and cultural rights. On the other hand, as Udin et al. [23], Walter [22] and Neves-Silva and Heller [24] pointed out, marginalized and excluded populations, who have their rights, among them HRWS, violated, have greater difficulty to participate in decision-making processes. Thus, one can see the importance of the concepts of indivisibility and interdependence of human rights.

It is important to draw attention to the study carried out by Leblang et al. [32] that demonstrated the positive intersection between democracy and economic freedom. It can be said, then, that economic-social vulnerability leads to the vulnerability of civil and political rights and reduced participation in democratic spaces. "Denial of economic freedom, in the form of extreme poverty, renders the person vulnerable to violations of other forms of freedom. [...]. The denial of economic freedom implies the denial of social and political freedom "Amartya Sen [33].

Therefore, as stated by Paul Farmer [34], human rights were created to protect the most vulnerable people. Hence, all documents drawn up on the basis of this framework will only be valid if they are used to protect the persons most at risk of having their rights violated.

One of the limitations of this study was the difficulty to interview some groups of homeless due to their alcohol and drug abuse. It is also important to consider that the findings in this study came from qualitative research, which was based, mainly, on what was reported by the participants. Therefore, they are more indicative than representative of the situation. Notwithstanding, important lessons could be drawn from the findings in this paper. In particular, the results of the study show how access to water and sanitation, in this context, is important in order to guarantee other rights. Future research could be done in other contexts to see if similar findings come out.

## Conclusions

The Economic, Social and Cultural covenant’s recognition impacts on a commitment to social integration, solidarity and equality, favoring the protection of vulnerable populations, so that basic needs are not conditional on charity and welfare programs, but are defined as a right. Within this set of rights are the HRWS. These rights, as discussed, are essential in securing other rights. Therefore, in order for us to talk about democracy and social participation, it is necessary that public policies turn towards vulnerable populations, respecting their particularities and specificities, and giving them opportunities and guaranteeing their rights.

Thus, for vulnerable populations to be truly contemplated, advances are needed in the conceptual expansion of human rights, incorporating the basic needs of social justice, and affirming the integral, indivisible and interdependent perspective that exists among them.

Therefore, it is important to affirm that the protection of human rights must be consolidated at an operational and normative level, aligned to concepts of indivisibility and interdependence as it has been proposed for approximately seven decades.

## Additional file

Additional file 1: Interview Guide. Questions made during the interviews. (DOCX 19 kb)

## Abbreviations

HRWS: Human Rights to Water and Sanitation
