# Supplementary material for: Human rights’ interdependence and indivisibility: a glance over the human rights to water and sanitation
Source: BMC Int Health Hum Rights. 2019 Mar 8;19:14. doi: 10.1186/s12914-019-0197-3 (PMC6408851; doi:10.1186/s12914-019-0197-3)
Supplement: Supplementary file 1 — Interview Guide. Questions made during the interviews. (DOCX 19 kb) [file 12914_2019_197_MOESM1_ESM.docx]

**Interview Guide**

**Date:**

**Name:** ____________________________________________________________________________________

**Sex:** _____________________________________________

**Birth Date:** ________________________

**Job/Occupation:** _____________________________________________

**Education level:** _________________________________________

**Civil State:** _____________________________________

**General Questions:**

How long have you been living on the street?

Why did you live on the street?

Why do not you stay in the public shelter?

How is your day to day life? Tell us a little bit about your life on the street.

Where do you find food?

What do you think about living on the street?

Do you live alone?

Do you have friends at the street?

Where do you look for support when you need?

Do you have access to health services when in need?

Have you lived in many places in the city?

Do you notice a lot of violence on the street? Have you ever suffered violence?

Do you want to leave the streets?

What does being on the street mean to you?

Do you have any source of income?

Do you feel part of the city and the society?

**Questions about access to water:**

What does water mean to you?

Do you feel that access to water on the street changed since you came to live here? (increased/reduced/easier/harder)

How and where do you get water to drink, to take a shower and wash your clothes?

Do you store water? How? where?

Do you have to pay for the use of water? How much?

Do you think this water is clean? Does it smell or have a different color?

Are you the person that gets the water you are going to use?

Do you have to walk long distances to get water?

How many times per day do you get water?

Do you feel, or ever felt, some fear when going to get water? When? Why?

Do you wash your clothes and utensils? Where?

Where do you take bath? How many times a week?

Do you have any idea of ​​how much water do you use per day?

Do you think that the amount of water you use daily is enough to drink, to do your personal hygiene and to clean your belongings?

Do you think the access to water you have today affects your health in any way? How?

Do you think access to water could improve? How?

**Questions about access to sanitation:**

How do you urinate and defecate?

Can you urinate and defecate whenever you want? Or need to wait for some specific time of the day to do your necessities?

Do you have privacy?

How do you clean yourself after going to the bathroom?

How do you clean yourself on menstruation days?

Have you ever had any diseases related to the lack of access to sanitation and hygiene?

Do you know if there are cases of violence against women when they need to urinate and defecate?

Do you feel or have you ever felt any fear when going to urinate or defecate? When? Why?

Do you think the access to sanitation that you have today affects your live and your health in any way? How?

Do you think the access to sanitation could improve? How?

**Questions About Human Rights:**

Do you think you have good access to water and sanitation?

Do you know that access to water and sanitation are human rights?

Do you participate or have already participated in any discussion on water and sanitation services?

Have you participated in the control of government actions and decisions regarding access to water and sanitation?

Do you know what the government and other actors (institutions) have done about the access to water and sanitation for homeless?
